# Potentiation of Imipramine-Induced Anti-hyperalgesic and Anti-Nociceptive Effects by Citicoline in the Sciatic Nerve Ligated Mice

**DOI:** 10.34172/aim.28772

**Published:** 2024-08-01

**Authors:** Negar Raissi-Dehkordi, Nastaran Raissi-Dehkordi, Bardia Hajikarimloo, Fatemeh Khakpai, Moammad-Reza Zarrindast

**Affiliations:** ^1^School of Medicine, Shahid Beheshti University of Medical Sciences, Tehran, Iran; ^2^Department of Physiology, Faculty of Medicine, Tehran Medical Sciences, Islamic Azad University, Tehran, Iran; ^3^Cognitive and Neuroscience Research Center (CNRC), Tehran Medical Sciences, Islamic Azad University, Tehran, Iran; ^4^Department of Pharmacology, School of Medicine, Tehran University of Medical Sciences, Tehran, Iran; ^5^Iranian National Center for Addiction Studies, Tehran University of Medical Sciences, Tehran, Iran; ^6^Institute for Cognitive Science Studies (ICSS), Tehran, Iran

**Keywords:** Citicoline, Imipramine, Nerve-ligated Mice, Neuropathic pain

## Abstract

**Background:**

Peripheral neuropathic pain is a result of damage/illness of the peripheral nerves. The mechanisms caused by its pathophysiology are not completely understood.

**Methods:**

Imipramine is a tricyclic antidepressant that is sometimes used to treat neuropathic pain. Moreover, citicoline is considered a novel adjuvant for painful disorders such as neuropathic pain. So, a possible interaction between imipramine and citicoline on pain behavior was examined in nerve-ligated mice using tail-flick and hot plate tests.

**Results:**

The results indicated that induction of neuropathic pain by sciatic nerve ligation caused hyperalgesia in nerve-ligated mice. On the other hand, intraperitoneal (i.p.) administration of citicoline (50, 75, and 100 mg/kg), and imipramine (2.5 and 5 mg/kg) induced anti-hyperalgesic and anti-nociceptive effects in nerve-ligated mice. Furthermore, citicoline potentiated the anti-hyperalgesic and anti-nociceptive effects of imipramine when they were co-administrated in nerve-ligated mice. Interestingly, there was an additive effect between imipramine and citicoline upon induction of anti-hyperalgesic and anti-nociceptive effects in nerve-ligated mice.

**Conclusion:**

Therefore, it can be concluded that citicoline (as an adjuvant substance) enhanced the efficacy of imipramine for the modulation of pain behavior in nerve-ligated mice

## Introduction

 Injuries in the central or peripheral nervous system elicit neuropathic pain.^1-4^ The understanding of a particular pathophysiological variation that contributes to the induction and preservation of neuropathic pain may provide a basis for the development of novel analgesic treatments for this illness. In this context, exploring the neurobiology of neuropathic pain has been accelerated by developing animal models that reflect some components of human pain conditions.^5-7^ Chronic constriction damage induced through ligation of the sciatic nerve has been widely used to clarify the pathophysiology of peripheral neuropathic pain.^8-12^ Ligation of the sciatic nerve in mice is a relevant model for assessing nociceptive and emotional consequences of continued neuropathic pain.^13,14^ Although neuropathic pain often does not react to conservative analgesic therapies, non-conservative painkillers such as antidepressants can be effective.^15^

 Antidepressants are often considered co-analgesics in chronic pain. Imipramine is a tricyclic antidepressant that is occasionally used to treat chronic neuropathic pain.^16-19^ The mechanism of action of imipramine in the improvement of neuropathic pain remains unclear, though it is prescribed as a robust reuptake inhibitor of serotonin and, to a lesser amount, norepinephrine.^18,20^ The mechanism is probable to vary from that in depression because analgesia with tricyclic antidepressants is frequently achieved at lower doses than required for antidepressant effects.^18^ Imipramine might induce an anti-nociceptive effect via activation of the serotonergic system.^21^ Imipramine may be administered either alone or in combination with other drugs for the treatment of neuropathic pains. For example, the cholinergic system is involved in the anti-nociceptive effect of imipramine.^22,23^

 Citicoline (cytidine 5’-diphosphocholine) as a dietary supplement is a necessary precursor in the synthesis of phosphatidylcholine, a main membrane phospholipid.^24-26^ Citicoline potentiates acetylcholine production.^26^ It is usually present in all cells. Exogenous citicoline simply crosses the blood-brain barrier and enhances the amount of choline available for acetylcholine synthesis as well as helps in rebuilding membrane phospholipid stores after depletion.^27^ Citicoline prevents the neuronal phospholipid membrane breakdown and repairs the neuronal membrane after neuronal damage.^28^ Long-term treatment with citicoline increases endogenous mechanisms of neurogenesis and neuro-repair contributing to physical therapy and recovery.^29^ Citicoline is considered a novel adjuvant for painful conditions such as peripheral neuropathic pain and diabetic polyneuropathy.^30-32^ This study examined a probable interaction between imipramine and citicoline on the control of neuropathic pain in nerve-ligated mice.

 In the present study, we selected imipramine, a tricyclic antidepressant, and citicoline, a novel adjuvant, that might play a role in the modulation of neuropathic pain. A previous study has reported a cross-talk between imipramine and citicoline regarding the induction of anti-nociceptive effects.^33^ So, this research aimed to evaluate a possible interaction between imipramine and citicoline on the modulation of neuropathic pain in nerve-ligated mice.

## Materials and Methods

###  Animals 

 Experiments were performed on male NMRI mice (6-8 weeks old, 20–25 g; Tehran University of Medical Sciences, Tehran, Iran) group-housed 3–4 per cage. The animals were maintained in a room with a controlled temperature of 23 ± 1 °C and a 12h/12h light/dark cycle (lights on 07:00 AM). Furthermore, food and water were available. Eight male mice were used for each experimental group. All experiments were done during the light period. The experimental techniques used in this research have been confirmed by the Ethics Committee of Tehran University of Medical Sciences (NIH publications No. 80-23).

###  Surgical Procedures 

 All surgeries were performed in sterile conditions and ketamine/xylazine anesthesia drugs (ketamine 50 mg/kg and xylazine 4 mg/kg, intraperitoneal (i.p.) injections). The common fiber of the right sciatic nerve was opened. Then, a 2-mm split section of the polyethylene tube was located near it. This induced an ipsilateral thermal hyperalgesia and a sustained ipsilateral mechanical allodynia.^13^ The shaved skin level was closed by a suture. The sham group underwent a similar surgery but without dissection of the sciatic nerve. The control group was only anesthetized.

###  Drug Treatment

 We used 0.9% saline for the control injection and to dissolve all drugs. Imipramine (Ciba-Geigy, Switzerland) and citicoline sodium (Minoo, Tehran, Iran) were used in this investigation. All drug injections were done intraperitoneally (i.p.; 10 mL/kg).

###  Anti-nociception Measurement

####  Tail-Flick Test

 A tail-flick apparatus was used to evaluate the nociceptive response to thermal stimulation (Borj Sanat Company, Iran). Each animal was slightly wrapped in a soft towel and the dorsal surface of the tail from its distal end was located in the apparatus every 15 minutes (for 60 minutes ) after the drug/saline administration. The heat source and a timer were started rapidly via a pedal. Both were ended automatically by a tail movement, which exposed a photocell under the tail or by the experimenter at the end of a 10-second cut-off time. The cut-off time was set to avoid skin damage. The response time between the start of the heat stimulus and the removal of the tail from the heat source was recorded via a sensor as the tail-flick latency. Individual tail withdrawal latency was changed to the percentage of maximum possible effect (%MPE) by this formula: %MPE = [(test latency-baseline latency)/ (cut off latency-baseline latency)] × 100. For all data, the area under the curve (AUC) of %MPE vs. time was recorded from 0 to 60 min by the trapezoidal rule to analyze the overall magnitude and time of the effect for the tail-flick test.

####  Hot-Plate Test 

 Pain sensitivity in sciatic nerve ligated mice was also evaluated via a hot-plate test as clarified in prior research.^34^ For this test, mice were placed on a 52 ± 0.2 °C heated plate (Borj Sanat Company, Iran). The time to lick the forepaw, hind paw, or jump was measured. We prescribed a cut-off time of 60 s to avoid any tissue damage.

###  Experimental Design 

 This research consisted of four experiments. In experiment 1, the effect of sciatic nerve ligation on tail-flick latency and hot-plate latency was examined. In experiment 2, the influence of saline (10 mL/kg), and diverse dosages of citicoline (25, 50, 75, and 100 mg/kg, i.p.) on tail-flick and hot-plate latencies were assessed. In experiment 3, the effects of injection of saline alone (10 mL/kg) or diverse dosages of imipramine (1.25, 2.5, and 5 mg/kg, i.p.), as well as co-administration of diverse dosages of imipramine (1.25, 2.5, and 5 mg/ kg, i.p.) along with a low dose of citicoline (25 mg/kg; i.p.) were evaluated in the tail-flick and hot-plate tests. In experiment 4, the effects of co-injection of imipramine 2.5 mg/kg + citicoline 50 mg/kg, and imipramine 1.25 mg/kg + citicoline 25 mg/kg, as well as imipramine 0.625 mg/kg + citicoline 12.5 mg/kg on pain-associated behaviors were examined. We used diverse groups of animals for the assessment of tail-flick and hot-plate latencies and each animal was only used for one test. Ten minutes after drug injection, tail-flick, and hot-plate tests were done. Table 1 explains the experimental groups.

**Table 1:** Experimental Groups.

**Figure**	**Panel**	**Drug Treatments (i.p.)**	**Effect on Tail-Flick**	**Effect On Hot-Plate**
1	A	Control, sham, and sciatic nerve ligated (saline, 10 mL/kg)	Hyperalgesia	-
	B	Control, sham, and sciatic nerve ligated (saline, 10 mL/kg)	Hyperalgesia	-
	C	Control, sham, and sciatic nerve ligated (saline, 10 mL/kg)	-	Hyperalgesia
2	A	Saline (10 mL/kg), citicoline (25, 50, and 100 mg/kg)	Anti-nociceptive	-
	B	Saline (10 mL/kg), citicoline (25, 50, and 100 mg/kg)	Anti-nociceptive	-
	C	Saline (10 mL/kg), citicoline (25, 50, and 100 mg/kg)	-	Anti-nociceptive
3	A	Saline (10 mL/kg), imipramine (1.25, 2.5, and 5 mg/kg)	Anti-nociceptive	-
	B	Saline (10 mL/kg), imipramine (1.25, 2.5, and 5 mg/kg) + citicoline (25 mg/kg)	Anti-nociceptive	-
	C (Left panel)	Saline (10 mL/kg), imipramine (1.25, 2.5, and 5 mg/kg)	Anti-nociceptive	-
	C (Right panel)	Saline (10 mL/kg), imipramine (1.25, 2.5, and 5 mg/kg) + citicoline (25 mg/kg)	Anti-nociceptive	-
	D (Left panel)	Saline (10 mL/kg), imipramine (1.25, 2.5, and 5 mg/kg)	-	Anti-nociceptive
	D (Right panel)	Saline (10 mL/kg), imipramine (1.25, 2.5, and 5 mg/kg) + citicoline (25 mg/kg)	-	Anti-nociceptive
4	A	Imipramine 2.5 mg/kg + citicoline 50 mg/kg Imipramine 1.25 mg/kg + citicoline 25 mg/kg Imipramine 0.625 mg/kg + citicoline 12.5 mg/kg	Additive anti-nociceptive	-
	B	Imipramine 2.5 mg/kg + citicoline 50 mg/kg Imipramine 1.25 mg/kg + citicoline 25 mg/kg Imipramine 0.625 mg/kg + citicoline 12.5 mg/kg	-	Additive anti-nociceptive

###  Statistical Analysis

 Normal distribution of data was examined using the Kolmogorov–Smirnov test. The homogeneity of variances was assessed using Levene’s test. The data were presented as mean ± standard error (SD) and 95% confidence interval (CI). The statistical analysis was done by one-way ANOVA and two-way ANOVA as well as Tukey’s post hoc test. The data of tail-flick test were examined via two-way ANOVA and repeated measures. In the repeated measures ANOVA, normality and homogeneity of variance of residuals as well as sphericity were assessed. A *P* value less than 0.05 was considered for significant difference.

 Furthermore, an isobolographic test was performed to define the interaction after the injection of two drugs. For this, the ED50 of each agent (2.5 mg/kg for imipramine and 50 mg/kg for citicoline) was examined via linear regression test and co-treatment of two drugs was administrated in fixed dosage ratio upon the ED50 amount. For drug co-treatment, theoretic ED50 is imipramine ED50/2 + citicoline ED50/2. Additionally, the experimental amount of drug combinations from the constant quantity determined was evaluated via the regression test (IBM SPSS Statistics, version 27 [IBM; Armonk, New York, USA]), after the experimental ED50 amount of the drug combinations was recorded (50% tail-flick and hot-plate latencies). The statistical significance between the theoretical ED50 and experimental ED50 of the drug co-treatment was recorded via a one sample t-test. If experimental ED50 was significantly lower than theoretical ED50, a synergistic interaction between imipramine and citicoline could be detected. Nevertheless, there was no difference between them showing additive interaction.^33^ Differences with *P *values less than 0.05 among experimental groups at each level were determined as statistically different.

## Results

###  Effect of Sciatic Nerve Ligation on Tail-flick and Hot-Plate Latencies in Male Mice 

 The results were considered as mean difference estimates with 95% CI. Table 2 displayed the results of mean ± standard error and 95% CI.

**Table 2:** Results of Mean ± Standard Error and 95% CI

**Figure**	**Panel**	**Statistical Analysis**	**Mean**	**Standard Error**	**95% Confidence Interval**	
					**Lower Bound**	**Upper Bound**
1	A	Two-way ANOVA	30.328	-	-	-
	B	One-way ANOVA	150.42	30.158	92	394
	C	One-way ANOVA	8.135	4.714	4	12
2	A	Two-way ANOVA	50.863	-	-	-
	B	One-way ANOVA	400.23	90.597	160.836	680.365
	C	One-way ANOVA	9.812	3.723	8.729	10.895
3	A	Two-way ANOVA	32.385	-	-	-
	B	Two-way ANOVA	45.202	-	-	-
	C (Left panel)	One-way ANOVA	200.52	35.591	180.397	450.397
	C (Right panel)	One-way ANOVA	214.23	57.394	180.397	502.364
	D (Left panel)	One-way ANOVA	9.650	3.813	8.430	10.869
	D (Right panel)	One-way ANOVA	14.737	5.654	12.929	16.545
4	A	One sample t-test	7.208	2.484	1.082	3.180
	B	One sample t-test	11.625	6.845	1.901	3.711

**Figure 1:**
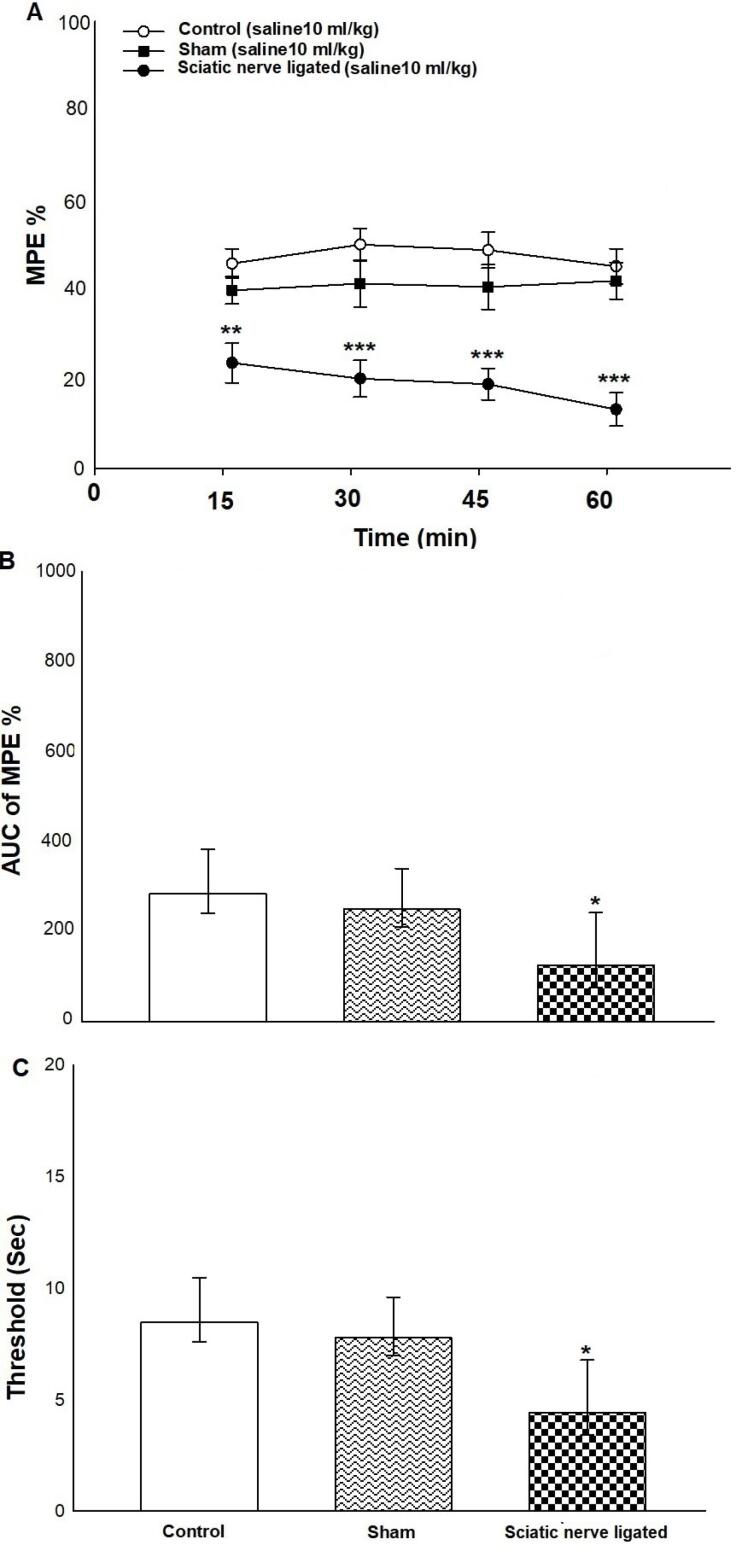
Influence of Sciatic Nerve Ligation on Pain Behavior in the Tail-Flick and Hot-Plate Latencies. Data are presented as mean ± SEM. Two-way ANOVA and repeated measures were performed for analysis of MPE% of the tail-flick test (A). One-way ANOVA and Tukey's post hoc test were performed for AUC of MPE% analysis (B) and hot-plate threshold (C). ^*^P < 0.05, **P < 0.01, and ^***^P < 0.001 in comparison to the control group (n = 8)

Figure 1 indicates the effect of sciatic nerve ligation on tail-flick latency and hot-plate latency inmale mice. Two-way ANOVA displayed no significant difference between sciatic nerve ligation and time intervals on %MPE [(time intervals effect F (1, 48) = 10.381, *P* < 0.001; sciatic nerve ligation effect F (2, 48) = 0.999, *P* = 0.400; time intervals × sciatic nerve ligation interaction F (2, 48) = 0.629, *P* = 0.599; Figure 1A]. About the time interval effect and sciatic nerve ligation effect, Tukey’s test showed that sciatic nerve ligation at the time intervals of 15, 30, 45 and 60 minutes increased %MPE innerve-ligated mice in comparison with the control and sham groups.

 One-way ANOVA and Tukey’s post hoc test for normalized AUC values showed that sciatic nerve ligation increased the AUC of %MPE innerve-ligated mice in comparison with the control and sham groups (mean difference: 243 and 95% CI: 92–394) [F (2, 21) = 3.437, *P*= 0.050; Figure 1B].

 Moreover, one-way ANOVA and Tukey’s *post-hoc* test showed a significant effect of sciatic nerve ligation on hot-plate latency (mean difference: 8 and 95% CI: 4–12) [(F (2, 21) = 3.776, *P* = 0.045; Figure 1C] in comparison with the control and sham groups. These data demonstrated that sciatic nerve ligation produced obvious hyperalgesia.

###  Effect of Citicoline on Neuropathic Pain in Nerve-Ligated Mice 

**Figure 2:**
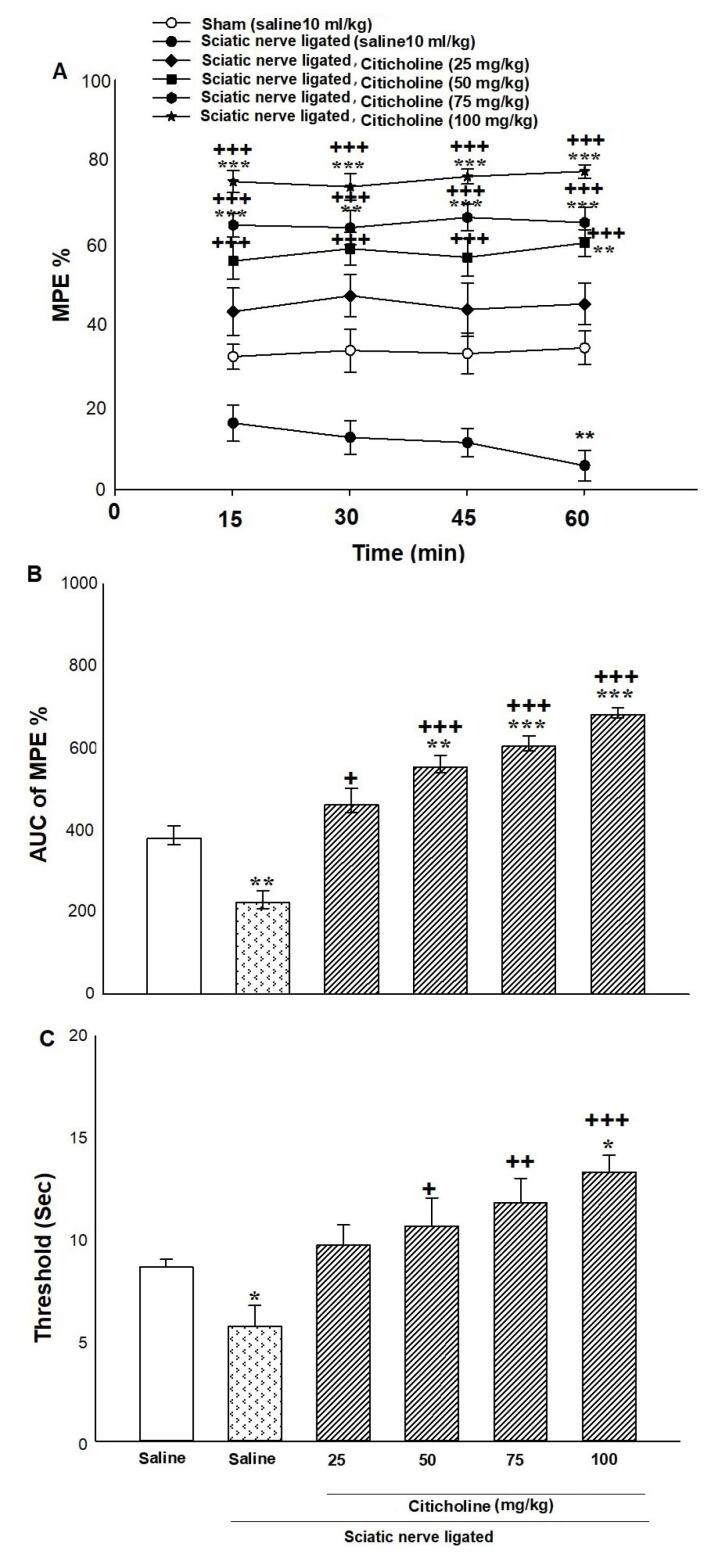
Influence of Diverse Doses of Citicoline (25, 50, 75, and 100 mg/kg) on the Pain Behavior of Nerve-Ligated Mice Investigated in the Tail-Flick and Hot-Plate Tests (each drug was administered to nerve-ligated mice). Data was presented as mean ± S.E.M. Two-way ANOVA and repeated measures were performed for analysis of MPE% of the tail-flick test (A). One-way ANOVA and Tukey’s *post-hoc* test were performed for AUC of MPE% analysis (B) and hot-plate threshold (C). ^*^P< 0.05, ^**^P < 0.01 and ^***^P < 0.001 in comparison to control group. ^+^ P < 0.05, ^+ +^ P < 0.01, and ^+ + +^*P* < 0.001 in comparison to nerve-ligated group (n = 8)

Figure 2 showed the effect of citicoline on tail-flick and hot-plate tests innerve-ligated mice. Two-way ANOVA displayed no significant interaction between citicoline dosages and time intervals on %MPE innerve-ligated mice [(time intervals effect F (1, 72) = 20.119, *P*< 0.001; citicoline effect F (5, 72) = 0.128, *P* = 0.942; time intervals × citicoline interaction F (5, 72) = 0.437, *P* = 0.727; Figure 2A]. About the time interval effect and citicoline effect, Tukey’s test exhibited that citicoline (50, 75, and 100 mg/kg; i.p.) at the time intervals of 15, 30, 45 and 60 minutes after administration increased %MPE innerve-ligated mice in comparison with the control and sham groups.

 Furthermore, one-way ANOVA and Tukey’s post hoc test for normalized AUC values showed that citicoline (50, 75, and 100 mg/kg; i.p.) enhanced the AUC of %MPE innerve-ligated mice compared to the control and sham groups (mean difference: 420.6 and 95% CI: 160.836–680.365) [F (5, 42) = 3.328, *P* = 0.007; Figure 2B].

 As seen in Figure 1C, one-way ANOVA showed a significant effect of citicoline on hot-plate latency innerve-ligated mice (mean difference: 9.812 and 95% CI: 8.729–10.895) [(F (5, 42) = 9.037, *P*< 0.001; Figure 2C] compared to the control and sham groups. Tukey’s analysis demonstrated that injection of diverse dosages of citicoline (50, 75, and 100 mg/kg; i.p.) enhanced the pain threshold in comparison with the control and sham groups. These data suggested that citicoline induced anti-hyperalgesic and anti-nociceptive effects innerve-ligated mice.

###  Effect of Imipramine and Citicoline Co-injection on Neuropathic Pain in Nerve-Ligated Mice 

**Figure 3:**
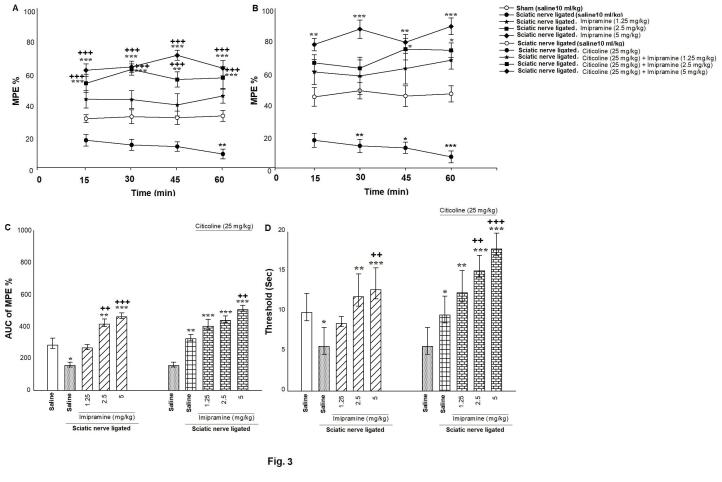
Influence of Administration of Imipramine Alone (1.25, 2.5, and 5 mg/kg) and Co-Administration of These Dosages Plus a Low Dose of Citicoline (25 mg/kg) on the Pain Behavior of Nerve-Ligated Mice Investigated in the Tail-Flick and Hot-Plate Tests (each drug was administered to nerve-ligated mice). Data are presented as mean ± S.E.M. Two-way ANOVA and repeated measures were performed for analysis of MPE% of the tail-flick test (A and B). One-way ANOVA followed by Tukey’s *post-hoc* test was performed for AUC of MPE% analysis (C) and hot-plate threshold (D). ^*^P < 0.05, ^**^P < 0.01 and ^***^P < 0.001 compared with control group. ^+ +^*P* < 0.01 and ^+ + +^*P* < 0.001 compared to nerve-ligated group (n = 8)

Figure 3A shows the effect of diverse dosages of imipramine (1.25, 2.5, and 5 mg/kg, i.p.) on tail-flick latency innerve-ligated mice. Two-way ANOVA analyses indicated no significant interaction between imipramine dosages and time intervals on %MPE innerve-ligated mice [(time intervals effect F (1, 64) = 19.973, *P*< 0.001; imipramine effect F (4, 64) = 18.935, *P*< 0.001; time intervals × imipramine interaction F (4, 64) = 0.150, *P* = 0.861; Figure 3A]. About the time interval effect and imipramine effect, Tukey’s test showed that imipramine (2.5 and 5 mg/kg; i.p.) at the time intervals of 15, 45, and 60 minutes after administration enhanced %MPE.

Figure 3B exhibited the effect of co-administration of diverse dosages of imipramine (1.25, 2.5, and 5 mg/kg, i.p.) plus a low dose of citicoline (25 mg/kg; i.p.) on the tail-flick latency innerve-ligated mice. Two-way ANOVA analyses displayed no significant interaction between drug combination dosages and time intervals on %MPE [(time intervals effect F (1, 64) = 79.663, *P* < 0.001; drugs-administration effect F (4, 64) = 6.994, *P* < 0.001; time intervals × drugs-administration interaction F (4, 64) = 0.958, *P* = 0.389; Figure 3B)]. About the time interval effect and drugs-injection effect, Tukey’s test showed that co-treatment of imipramine and citicoline enhanced %MPE in tail-flick at the time intervals of 15, 30, 45 and 60 minutes after co-administration.

 Furthermore, one-way ANOVA and post hoc analysis showed a significant effect of diverse dosages of imipramine (2.5 and 5 mg/kg, i.p.) (mean difference: 315.397 and 95% CI: 180.397–450.397) [F (4, 35) = 7.922, *P* < 0.001; Figure 3C, left panel] as well as co-treatment of these doses plus a low dose of citicoline (25 mg/kg; i.p.) (mean difference: 341.38 and 95% CI: 180.397–502.364) [F (4, 35) = 9.877, *P* < 0.001; Figure 3C, right panel] on the AUC of %MPE innerve-ligated mice.

Figure 3D exhibited the effect of administration of diverse doses of imipramine alone (1.25, 2.5, and 5 mg/kg; i.p.) as well as co-injection of these doses along with a low dosage of citicoline (25 mg/kg, i.p.) on pain threshold in the hot plate latency. Pain threshold was enhanced by administration of imipramine alone (2.5 and 5 mg/kg, i.p.) (mean difference: 9.649 and 95% CI: 8.430–10.869) [One-way ANOVA followed by post hoc analysis: F (4, 35) = 6.854, *P*< 0.001; Figure 3D, left panel] as well as co-administration of this dose plus a low dose of citicoline (25 mg/kg; i.p.) (mean difference: 14.737 and 95% CI: 12.929–16.545) [One-way ANOVA followed by post hoc analysis: F (4, 35) = 21.827, *P*< 0.001; Figure 3D, right panel] in nerve-ligated mice. These results revealed that imipramine produced anti-hyperalgesic and anti-nociceptive effects innerve-ligated mice and citicoline potentiated imipramine response.

###  Additive Effect of Imipramine and Citicoline on Induction of Anti-nociceptive Effect

**Figure 4:**
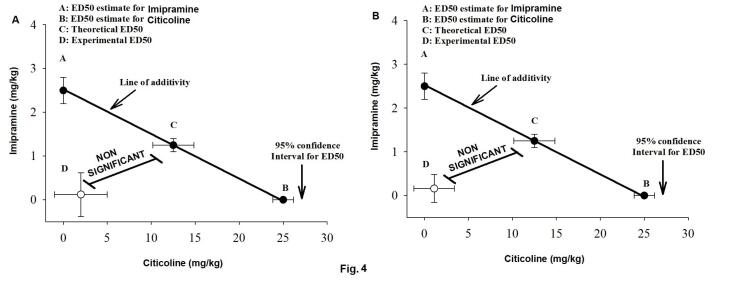
Isobologram Analysis of the Effects of Drug Treatment Indicates the Additive Influence of Imipramine and Citicoline on the Induction of Anti-hyperalgesic and Anti-nociceptive Effects in Nerve-Ligated Mice. Statistical analysis showed no significant difference between experimental ED50 and theoretical ED50 points, presenting an additive influence of the co-treatment of the drugs ((A) for tail-flick latency and (B) for hot-plate latency. ED50, effective dose 50

 The theoretical additive line (the regression test, IBM SPSS Statistics, version 27 [IBM; Armonk, New York, USA]) exhibited that the imipramine and citicoline co-treatment induced an effect on theoretical %50 tail-flick latency (Figure 4A) and theoretical %50 hot plate latency (Figure 4B) (theoretical ED50). One sample t-test showed that there was no significant difference between experimental ED50 and theoretical ED50 for the tail-flick latency (mean difference: 2.131 and 95% CI: 1.082–3.180) [T (23) = 0.667, *P* = 0.511; Figure 4A] and hot plate latency (mean difference: 2.806 and 95% CI: 1.901–3.711) [T (23) = 0.836, *P* = 0.726; Figure 4B]. Our data proposed an additive effect of imipramine and citicoline co-treatment on induction of anti-hyperalgesic and anti-nociceptive effects in nerve-ligated mice.

## Discussion

 Tail-flick and hot plate apparatuses are validated tests for the evaluation of pain. The difference between tail-flick and hot plate tests is that the tail-flick device assesses the nociceptive response mostly at the spinal level while the hot plate device assesses the supra-spinal response.^35^ Induction of neuropathic pain by ligation of the sciatic nerve is one of the best and most frequently used models of assessment of anti-hyperalgesic and anti-nociceptive properties of substances.^4,36^ Our research indicated that sciatic nerve ligation caused hyperalgesia in nerve-ligated mice in comparison with the control and sham groups. Studies demonstrated that partial ligation of the sciatic nerve caused a highly reproducible syndrome in the mice, including a reduction in thermal and mechanical nociceptive thresholds and sustained alterations in neurotransmitter and receptor expression.^7,12^ Consistent with some studies on neuropathic pain, we also demonstrated hyperalgesia in nerve-ligated mice.

 The current research showed that citicoline at doses of 50, 75, and 100 mg/kg reduced hyperalgesia. Here, citicoline dose-dependently induced anti-nociceptive effect following sciatic nerve ligation in mice. Citicoline has been demonstrated to prompt nerve regeneration after surgery in numerous *in vitro* investigations.^37^ Citicoline has been commonly used in clinical cases of central nervous system illnesses, for example, ischemic stroke, cognitive impairments, and glaucoma. Administration of citicoline has been revealed to play a role in improving motoric function and prompting the regeneration process of the impaired axons in a rat sciatic nerve damage model, showing its potential role in the treatment of peripheral nerve injury.^31,38,39^ According to our results, Kanat and colleagues indicated that citicoline exerted an anti-hyperalgesic effect in oxaliplatin-induced neuropathic pain.^32^ Also, Emril et al reported that citicoline administration prevented peripheral neuropathic pain after sciatic nerve crush injury in rats.^31^ Following administration of citicoline, it is hydrolyzed to cytidine and choline, which leads to enhanced plasma and tissue concentrations of these metabolites.^40^ Citicoline stimulates the biosynthesis of structural phospholipids of the neuronal membranes, raises brain metabolism, and acts upon the levels of diverse neurotransmitters, for instance, norepinephrine and dopamine. Owing to its weird pharmacological properties and action mechanisms, citicoline has been described as a potential candidate agent for the treatment of several types of neurological disorders such as head trauma, cerebral vascular disease, and Alzheimer’s disease.^41^ Recent preclinical investigations indicated that administration of citicoline elicited dose- and time-dependent anti-nociceptive and anti-hyperalgesic effects in behavioral models of neuropathic and inflammatory pain in rodents.^42,43^ These effects of citicoline might be mediated by an interaction between acetylcholine, norepinephrine, and dopamine receptors.^32^

 In the next section of this research, we assessed the effect of the administration of imipramine alone on the modulation of neuropathic pain induced by sciatic nerve ligation in male mice. Our data exhibited that i.p. injection of imipramine dose-dependently enhanced %MPE and AUC of %MPE inthenerve-ligated mice, showing anti-hyperalgesic and anti-nociceptive effects. Tricyclic antidepressants such as imipramine have analgesic effects in diverse chronic pain disorders that are distinct from their antidepressant characteristics.^19,44^ In experimental animals, numerous investigations also have reported that imipramine induced an anti-nociceptive effect on various nociceptive stimuli.^44-46^ Different doses of imipramine induced a dose-dependent anti-nociceptive effect in the formalin test,^23,46-49^ tail-flick method,^22,33^ and hot-plate test.^45^ The anti-nociceptive mechanism of antidepressants is still obviously detected. Imipramine may induce an anti-nociceptive effect via the serotonergic and cholinergic systems.^21-23^

 Tricyclic antidepressants are used either alone or in combination with other substances to treat neuropathic pains, so we decided to examine a possible interaction between imipramine and citicoline as well as to explore the significance of imipramine doses (1.25, 2.5, and 5 mg/kg) on the modulation of neuropathic pain in nerve-ligated mice. Our findings exhibited that co-treatment of different doses of imipramine (1.25, 2.5, and 5 mg/kg) along with an ineffective dosage of citicoline (25 mg/kg) increased %MPE and AUC of %MPE inthenerve-ligated mice, suggesting that citicoline potentiated the anti-hyperalgesic and anti-nociceptive effects of imipramine. Interestingly, our data revealed an additive effect between imipramine and citicoline upon induction of anti-hyperalgesic and anti-nociceptive effects in nerve-ligated mice. As mentioned previously, the cholinergic system is involved in the anti-nociceptive effect of imipramine.^22,23^ We proposed that citicoline increased acetylcholine levels,^26^ hence potentiated the antihyperalgesic and anti-nociceptive effects of imipramine. In this context, our previous research indicated that co-treatment of imipramine and citicoline induced an analgesic effect in intact male mice.^33^

## Conclusion

 The anti-hyperalgesic and anti-nociceptive effects of imipramine and citicoline might be due to the enhancement of some neurotransmitters such as serotonin, acetylcholine, and norepinephrine. Furthermore, the encouraging results of the animal experiment may prompt further clinical assessment of the effects of imipramine and citicoline in the management of neuropathic pain.
